# Silymarin and MSC-exosomes ameliorate thioacetamide-evoked renal fibrosis by inhibiting TGF-β/SMAD pathway in rats

**DOI:** 10.1007/s11033-024-09343-6

**Published:** 2024-04-18

**Authors:** Dina Mohamed Mekawy, Dina Sabry, Rania Mohamed Sabry, Naglaa F. Abozeid

**Affiliations:** 1 Medical Biochemistry and Molecular Biology Department, Kasr Al-Aini Faculty of Medicine, Cairo University, Cairo, 11956 Egypt; 2 Medical Biochemistry and Molecular Biology Department, Faculty of Medicine, Badr University in Cairo, Badr City, Egypt; 3 Department of Anatomic Pathology, Kasr Al-Aini Faculty of Medicine, Cairo University, Cairo, 11956 Egypt

**Keywords:** Thioacetamide, Silymarin, Exosomes, MSCs, TGF

## Abstract

**Background:**

TGF-β1 and SMAD3 are particularly pathogenic in the progression of renal fibrosis.

**Aim:**

This study aimed to evaluate the kidney protective potentials of silymarin (SM) and exosomes of mesenchymal stem cells against the nephrotoxin thioacetamide (TAA) in rats.

**Methods:**

32 female rats were randomly assigned into four groups: the control group, the TAA group, the TAA + SM group, and the TAA + Exosomes group. The kidney homogenates from all groups were examined for expression levels of TGF-β receptors I and II using real-time PCR, expression levels of collagen type I and CTGF proteins using ELISA, and the expression levels of nuclear SMAD2/3/4, cytoplasmic SMAD2/3, and cytoplasmic SMAD4 proteins using the western blot technique.

**Results:**

Compared to the control group, the injection of TAA resulted in a significant increase in serum levels of urea and creatinine, gene expression levels of TβRI and TβRII, protein expression levels of both collagen I and CTGF proteins, cytoplasmic SMAD2/3 complex, and nuclear SMAD2/3/4 (p-value < 0.0001), with significantly decreased levels of the co-SMAD partner, SMAD4 (p-value < 0.0001). Those effects were reversed considerably in both treatment groups, with the superiority of the exosomal treatment regarding the SMAD proteins and the expression levels of the TβRI gene, collagen I, and CTGF proteins returning to near-control values (p-value > 0.05).

**Conclusion:**

Using in vitro and in vivo experimental approaches, the research discovered a reno-protective role of silymarin and exosomes of BM-MSCs after thioacetamide-induced renal fibrosis in rats, with the advantage of exosomes.

**Supplementary Information:**

The online version contains supplementary material available at 10.1007/s11033-024-09343-6.

## Introduction

Despite the underlying etiology, renal fibrosis is the ultimate fate for chronic renal diseases [1]. Thioacetamide (TAA; CH3CSNH2) is a component of fungicides, pesticides, and pharmaceuticals. Severe nephrotoxicity, associated with TAA-induced oxidative stress, inflammatory response, and apoptosis, causes impairment of kidney function and fibrosis [2, 3]. Furthermore, it can produce hepatotoxic sulfur oxides and dioxides [4], though kidneys are more vulnerable to injury than other organs [5]. This study aimed to estimate the impact of SM and MSC exosomes on TAA-induced nephrotoxicity by shedding light on their role in TGF-β/SMAD signaling.

Silymarin (SM) is an herbal polyphenolic flavonoid complex extracted from an ancient plant [6]. It has been demonstrated to possess antioxidant and antiproteinuric effects on humans and animal models [7]. It has no side effects, even at a relatively high dosage [8]. Exosomes originating from mesenchymal stem cells (MSCs) are a subpopulation of extracellular vesicles that are nanosized (30–150 nm) and released into the extracellular space, playing a biological role in the transmission of a mixture of cargos, including proteins, lipids, mRNA, and miRNA [9]. The mechanisms by which they exert their beneficial effects are many-sided, but in general, they act mainly by controlling Akt, mTOR, JAK2/STAT3, P38-MAPK, and TGF-β signaling [10]. Exosomes have different RNA and RNA binding proteins, which differ from that of the mother cell [11]. Superior to MSCs, exosome-based therapy avoids some of the issues and restrictions associated with using such live, reproducing cells [12].

Among many identified fibrogenic elements, transforming growth factor-β (TGF-β) has an orchestrated role in renal fibrosis and chronic kidney disease [13]. It consists of highly pleiotropic molecules; TGF-β1 is the most abundant in fibrosis. TGF-β signaling commences by binding to homodimeric TβRII (type II TGF-β receptor), which attracts and phosphorylates two units of TβRI. Then, the TβRI phosphorylates and activates its substrates, the suppressor of mothers against decapentaplegic (SMAD) [14] and non-SMAD pathways [15]. The phosphorylated SMAD2 and SMAD3 function in concert and form a hetero-oligomer with SMAD4 (Supplementary Information 1) to complete the SMAD4-mediated plasmid-nucleus shuttle to control the expression of some genes [16]. Such action induces the production of excessive extracellular matrix (ECM), including collagen I (through binding of collagen promoter COL1A2), integrins, and fibronectin, or suppresses ECM degradation by inhibiting matrix metalloproteinases (MMPs), helping endothelial-mesenchymal transition (EMT). Many of these fibrotic destructive events are caused by the profibrotic effects of SMAD3 rather than SMAD2. SMAD2 seems to have a renoprotective, anti-fibrotic role [17]. Collagen-1 was profoundly suggested to be involved in the pathological progression of renal fibrosis and the unnecessary accumulation of ECM [18]. Connective tissue growth factor (CTGF) is one of the matricellular proteins, the most abundant in fibrotic kidney scaffolds. CTGF, as a TGF-β downstream effector, can prompt fibroblast activation, especially in glomerular mesangial cells [19]. As targeting TGF-β is likely to have unexpected adverse effects [20], it is necessary to focus on individual TGF-β activation steps more localized to reduce overall toxicity [15].

## Materials and methods

### Experimental design for in vivo treatments

A total of 34 mature albino rats of the Wistar strain, weighing 150–200 g, aged 5–7 months, two male and 32 female rats, were maintained along with the standards of the Institutional Animal Care and Use Committee (IACUC), Faculty of Medicine, Cairo University (CU-III-F-4-23). They came from the animal shelter, Faculty of Medicine, Cairo University. All animals were adapted two weeks before the experiment. TAA (98%) and SM (80% purity) were obtained from Sigma-Aldrich (Saint Louis, MO, United States). All the chemicals were of the highest analytical grade.

The two male rats were used as donors for exosomes of BM-MSCs to allow detection of homing of the Y chromosome into the kidney tissue of the female rats.

Four groups of eight female rats were formed by randomly dividing the female rats as follows: The control group received 1 ml of saline intraperitoneally twice weekly for six weeks. The TAA group (the positive drug control group) received thioacetamide (150 mg/kg, dissolved in normal saline) via intraperitoneal (i.p.) injection twice weekly for six weeks. The TAA + SM group received the dosage of TAA administered intraperitoneally with silymarin (50 mg/kg/day) by oral gavage for six weeks. The TAA + Exosomes group received i.p. shots of TAA (as described in the TAA group) preceded by a single IV injection of previously prepared exosomes of BM-MSCs (4 µg/ml PBS) per rat via tail vein. A few days after the final treatment, a total volume of 0.5 mL of blood was drawn from the tail tip vein of each rat once. Afterward, all animals were euthanized by cervical dislocation under diethyl-ether anesthesia (1.9%), and then laparotomy was performed, and the two kidneys were extracted.

The right kidney was washed and separated into two pieces. One piece was submerged in 10% neutral buffered formalin for 72 h to evaluate the morphological alterations, and the other was processed for immunoblotting. After homogenization and centrifugation of the left kidney, the supernatant retrieved was divided into aliquots and kept at − 80^o^C pending RNA expression, RT-PCR, and ELISA. A summarized methodology can be obtained from (Supplementary Information 2).

### Culture of bone marrow-extracted mesenchymal stem cells (BM-MSCs)

The tibiae and femurs of the two male rats were used as the source of the primary BM-MSCs. Bone marrow was harvested using Dulbecco’s modified Eagle’s medium (DMEM, GIBCO/BRL). Propagation of cells was allowed in 95% air and 5% CO2 at 37 °C until > 90% confluence using a medium of DMEM enriched with 10% fetal bovine serum, 100 U/mL penicillin, and 100 µg/mL streptomycin (Gibco, Thermo Fisher, Scientific, Inc.). The medium was replaced every 2–3 days to maintain cell viability. Then, the cells were detached after trypsinization with a 0.25% trypsin-EDTA solution (Gibco, Thermo Fisher, Scientific, Inc.), and subcultures were prepared to continue proliferation. In the fourth passage, the potential capabilities of the BM-MSC differentiation were assessed as follows: for adipogenic differentiation, this research used the StemPro® adipogenic differentiation kit (Gibco, Life Technology, Carlsbad, CA, USA), and they were stained with Alcian blue (Sigma-Aldrich, St. Louis, MO, USA). For osteogenesis differentiation, the StemProⓇ kit (Gibco, Life Technologies) and Alizarin Red S stain (Sigma-Aldrich) were used. After the seventh passage, the culture supernatant was separated to isolate the exosomes.

### Isolation of BM-MSC exosomes

BM-MSC-exosomes were extracted according to a previously described protocol [21]. Briefly, in a centrifuge, the culture supernatant eliminated the cell debris. Then, the exosome pellets were harvested by ultracentrifugation (Beckman-Coulter Optima L-90 K). Finally, the exosome pellet was stored at -80 °C. The exosome morphology was examined using a transmission electron microscope (TEM, HT-7700, Hitachi, Japan).

### Biochemical analysis of kidney function tests

Sera were collected by centrifuging the blood specimens after clotting and kept at -20 °C until the measurement of urea and creatinine levels was later performed using the commercial packs of Diamond Diagnostics, Cairo, Egypt.

### Histopathological examination

The kidney tissues were examined grossly before being processed automatically in a tissue processor, which included the following steps: ethanol-based dehydration, xylene-based clearing, and paraffin wax block final embedding.

The manual microtome was cut into 4–5 micron sections and then stained with hematoxylin-eosin (H&E) [22]. Masson’s trichrome was used to demonstrate collagen fibers in the foci of fibrosis [23]. Slides were evaluated blindly using light microscopy (Leica DM1000), and representative lesions were captured.

### Gene expression measurement by real-time PCR (RT-PCR)

The kidney homogentisate was assayed for mRNA expression of TβRI and TβRII by RT-PCR against β-actin as a housekeeping gene. RNA was harvested using TRIzol® (Invitrogen, Carlsbad, CA, USA) and reverse transcribed using PrimeScript RT Master Mix (Takara Bio, Japan) as per instructions provided by the manufacturers. The sequences of the primers are listed in Table 1. Gene expression was estimated using the ΔΔCT method normalized against β-actin.

**Table 1:** Primers sequence of the studied genes

Gene	5′ → 3′ Primmer sequence F: Forward primer, R: Reverse primer	Reference
β-Actin	F: TGCTATGTTGCCCTAGACTTCG R: GTTGGCATAGAGGTCTTTACGG	[24]
TβRI	F: TGGCGGAATCCACGAAGA- R: ACGGATGGATCAGAAGGTACAAG	[25]
TβRII	F: GGATGGCAAAGAGATAACCCA R: AGAGTGAAGCCGTGGTAGGTGAGCTT	[26]

### Quantitative gene expression using ELISA

The kidney homogentisate was used to measure the expression levels of collagen type I using the CUSABIO Col I ELISA Kit (#Cat No. CSB-E08084r), and CTGF proteins using the MyBioSource ELISA Kit (#Cat No. MBS261004), according to the manufacturer’s protocols.

### Western blotting analyses

The kidney tissues were homogenized in lysis buffer (Cell Signal Technology Inc., Danvers, MA) and centrifuged at 20,000 ×g for 60 min at 4 °C. One gram of tissue was weighed and homogenized in 5 ml of ice-cold 1X hypotonic buffer supplemented with 1 Mm DTT and 1% detergent solution. Centrifugation was performed for 15 min at 10,000 rpm at 4^o^C. The cytoplasmic fraction was transferred into a 15 ml tube and stored at 4^o^C. The pellet is the nuclear fraction. The nuclear pellet was suspended in a 500 µl nuclear lysis buffer by pipetting up and down. The suspension was centrifuged at 20,000 rpm for 45 min at 4^o^C in a microcentrifuge. The protein concentration in the nuclear extract was evaluated using a detergent-compatible assay technique (e.g., BioRad DC Protein Assay Method). Protein concentrations were determined using a Bio-Rad protein assay. Specific proteins were then incubated with their corresponding primary antibodies (nuclear SMAD2/3/4 (OABB00952, Aviva Systems Biology, USA), cytoplasmic SMAD2/3 (Santa Cruz Biotechnology, Inc. sc-133,098, Europe), and cytoplasmic SMAD4 (Santa Cruz Biotechnology, Inc. sc-7966, Europe) in TBS-T with 2% BSA for two hours at room temperature. β-actin has been used as the loading control for relative band density analysis. Membranes were subjected to successive steps of washing, incubation with IgG antibodies, and washing for 60, 30, and 60 min, respectively, using Tris-NaCl-Tween (TNT) clean buffer and horseradish peroxidase-conjugated goat anti-rabbit IgG (1:5000). For visualizing immunoreactivity, chemiluminescence (ECL) and western blotting detection reagents (Amersham Biosciences, Amersham, Buckinghamshire, UK) were used.

### Statistical analysis

All data were presented as the means ± SD (*n* = 8). The SigmaStat software (Jandel Scientific), one-way analysis of variance (ANOVA), and Dunnett’s post-hoc multiple comparison tests were used to determine the statistical significance. A statistically significant difference was determined to exist when the p-value was less than 0.05.

## Results

### BM-MSCs isolation and collection of their exosomes

**Fig. 1:**
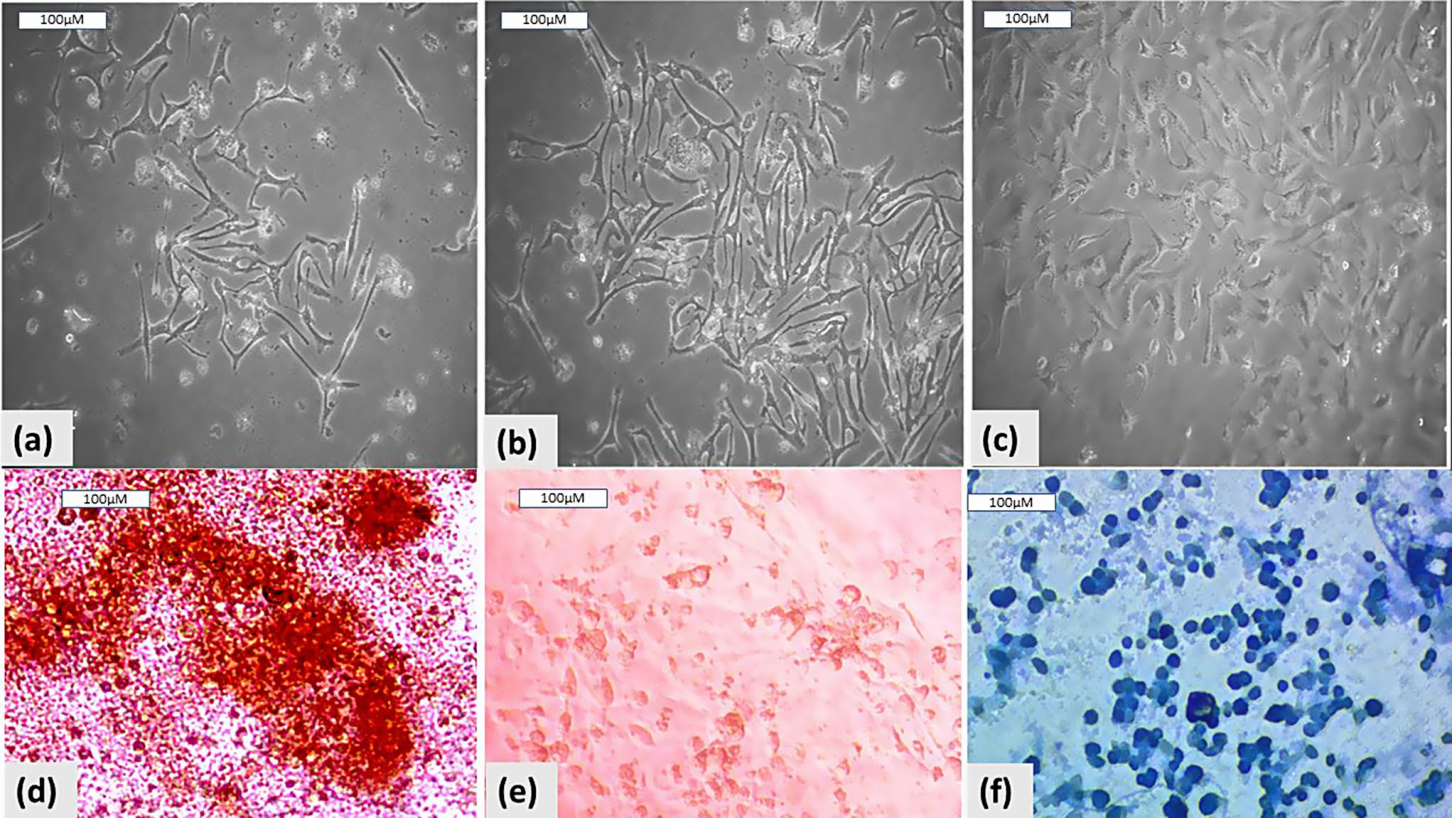
Differentiation of the isolated BM-MSCs. BM-MSCs at days 7 (**a**), 14 (**b**), and 21 (**c**). Alizarin red staining of BM-MSC differentiation shows their osteogenesis. (**d**), Oil Red O staining of BM-MSC differentiation shows their adipogenesis (**e**), and Alcian blue staining of BM-MSC differentiation shows their chondrogenesis (**f**)

**Fig. 2:**
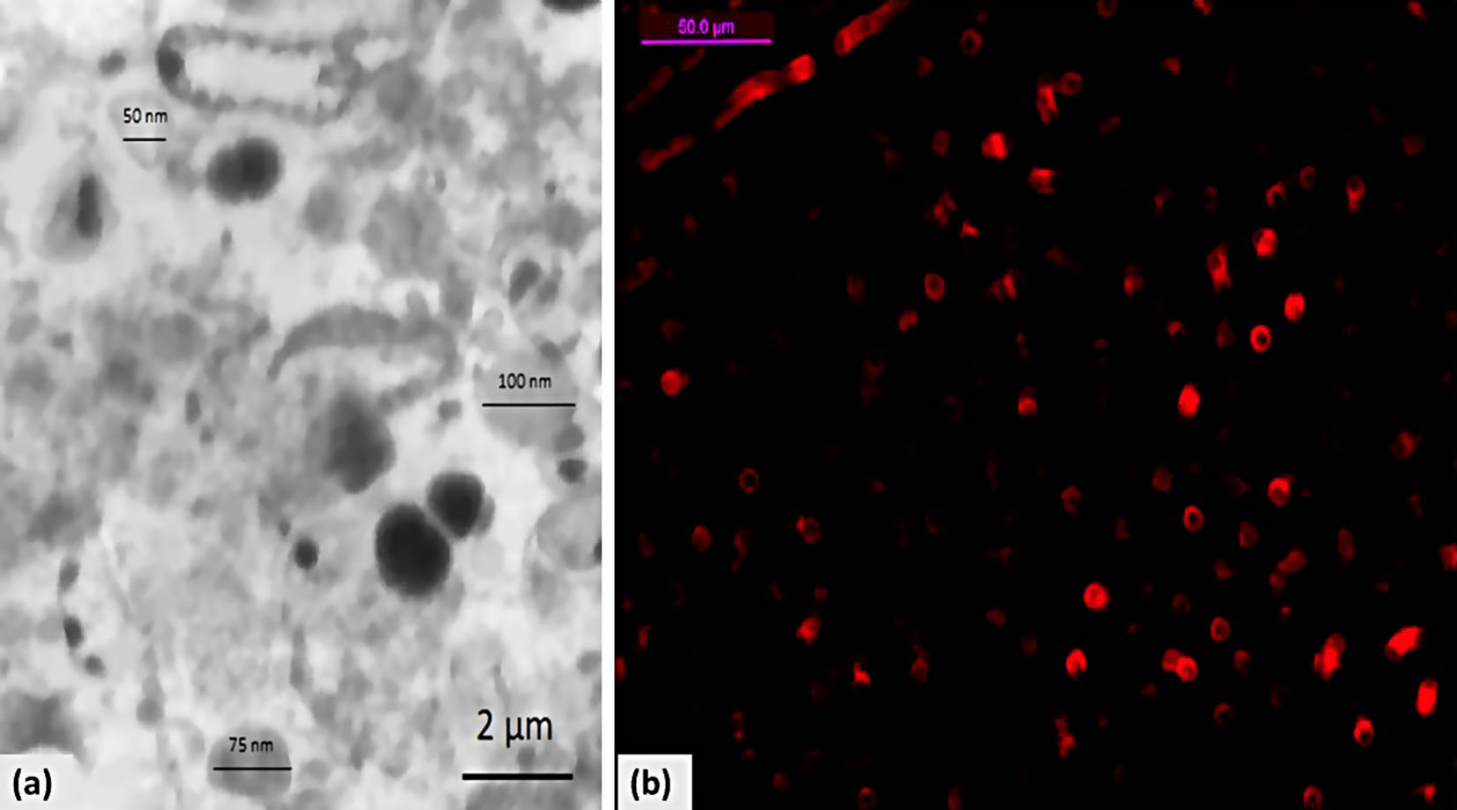
(**a**) Morphology of the isolated BM-MSC-exosomes (TEM); (**b**) Homing of BM-MSCs-derived exosomes in the kidney tissue

BM-MSCs were distinguished by their usual plastic adherence, fibroblast-resembled morphology (Fig. 1a, b and c), and their differentiation capabilities: osteogenic, adipogenic, and chondrogenic (Fig. 1d, e, and f). The derived exosomes can be seen in Fig. 2a, and their homing is shown in Fig. 2b.

### Serum levels of urea and creatinine

The injection of TAA, making renal fibrosis supervenes, resulted in a relentlessly significant increase in urea and creatinine serum levels (p-value < 0.001). The administration of SM and injection of exosomes significantly decreased their serum levels (p-value < 0.001) (Table 2).

**Table 2:** Serum levels of urea and creatinine in the studied groups

	Control (*n* = 8)	TAA (*n* = 8)	TAA + SM (*n* = 8)	TAA + Exosomes (*n* = 8)
Urea (mg/dl)	23.3 ± 1. 35	56.23 ± 1.97	30.22 ± 1.02	33.5 ± 0.32
Creatinine (mg/dl)	0.61 ± 0.03	1.74 ± 0.02	0.98 ± 0.23	0.92 ± 0.14

### Histopathological alterations of the kidneys

**Fig. 3:**
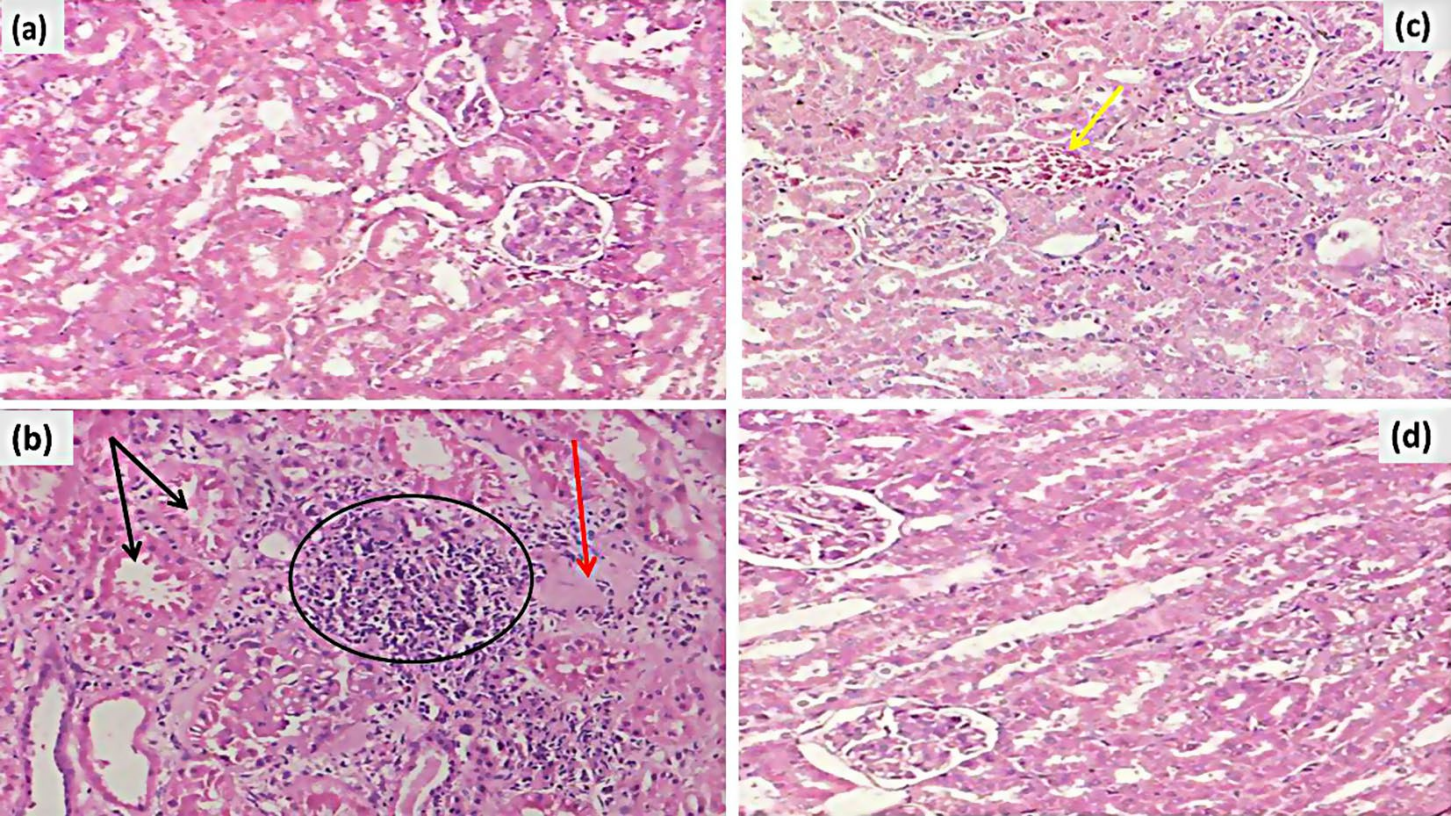
(Histopathological alterations of the kidney tissues (H&E). (**a**) Control group: healthy kidney tissue (x400); (**b**) TAA group: interstitial fibrosis (red arrow), tubular injury (black arrow), and chronic inflammatory infiltrate (black circle) (x400); (**c**) TAA + SM group: There is congestion (yellow arrow) with residual tubular injury and minimal residual fibrosis after treatment (x400); and (**d**) TAA + Exosomes group: There is a significant improvement in the treated group (x400)

**Fig. 4:**
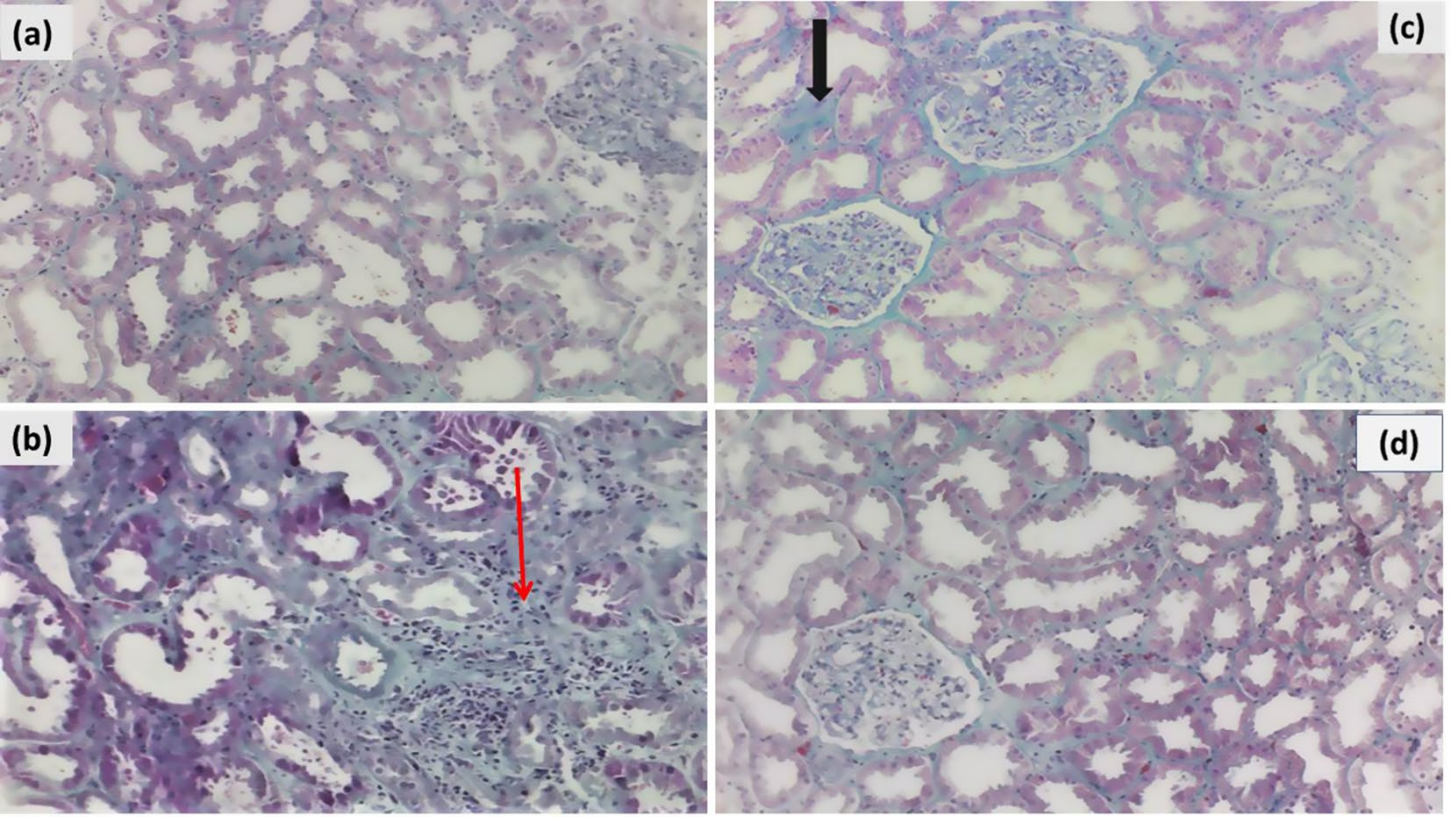
Histopathological alterations of the kidney tissues (Masson’s trichrome stain). (**a**) Control group: healthy kidney tissue (x400); (**b**) TAA group: interstitial fibrosis (red arrow) and peritubular fibrosis (x400); (**c**) TAA + SM group: there is some improvement, but with residual minimal fibrosis (black arrow) (x400); (**d**) TAA + exosomes group: there is more evident improvement (x400)

The kidney prepared from the control group revealed a normal renal corpuscle with glomerular tuft capillaries, a double-walled Bowman’s capsule, and normal proximal and distal tubule-lined tubular epithelium (Figs. 3a and 4a).

The damage effects of TAA appear in the form of marked tubular injury, interstitial fibrosis, and chronic inflammatory infiltrates (Fig. 3b). Masson’s trichrome stain is highlighted, showing interstitial and peritubular fibrosis (Fig. 4b). Both groups treated by SM and exosomes reveal a reduction of fibrosis and chronic inflammatory infiltrates (Figs. 3c and d and 4c, and 4d), with more normalization of histomorphology and degenerative changes in the exosome group (Figs. 3d and 4d).

### RT-PCR expression levels of TGF-β receptors (TβRI and TβRII)

**Fig. 5:**
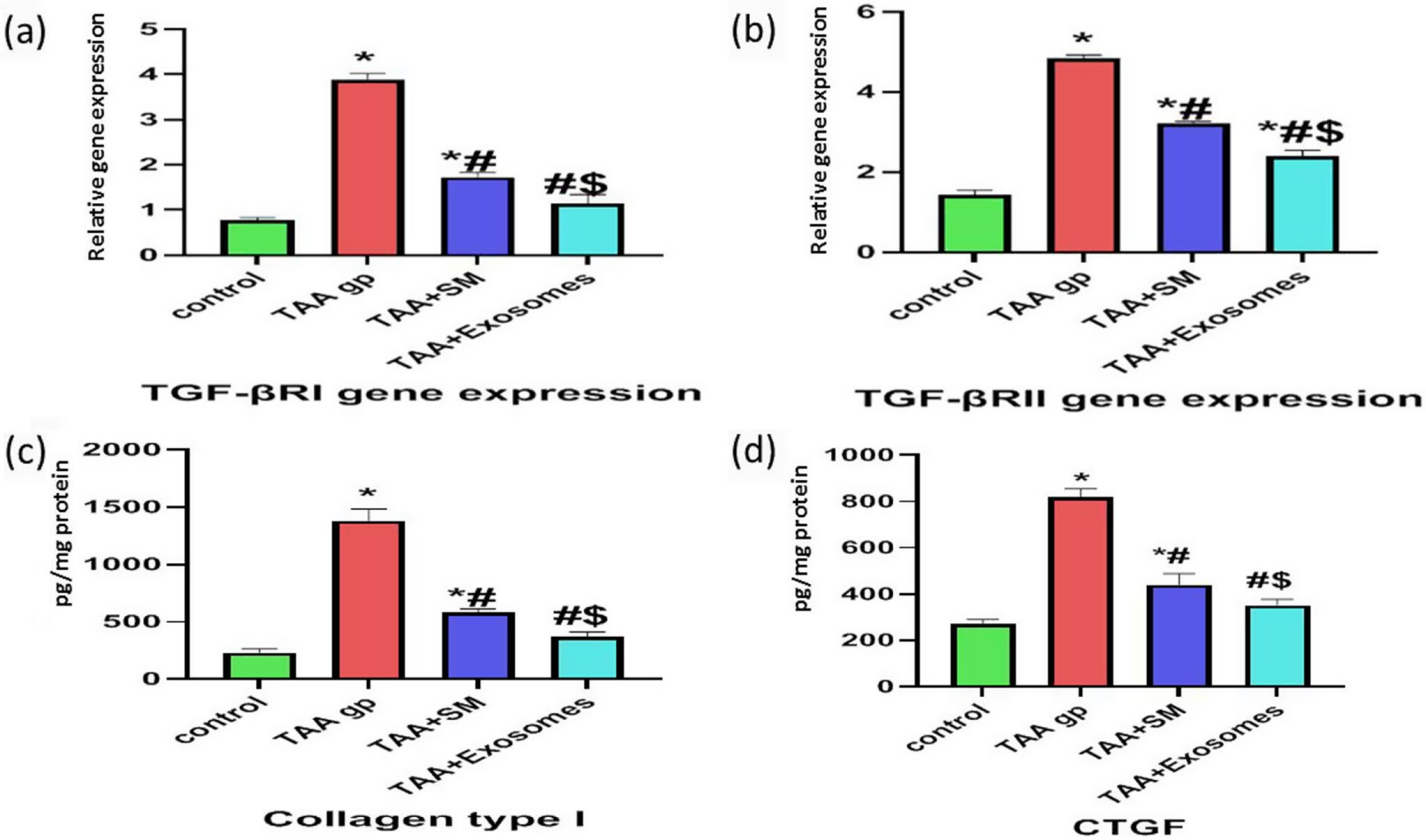
RT-PCR expression levels of TGF-β receptors and ELISA expression levels of the studied proteins. (**a**) RT-PCR expression levels of TβRI; (**b**) RT-PCR expression levels of TβRII. (**c**) ELISA expression levels of collagen type I protein; (**d**) ELISA expression levels of CTGF protein. Data are expressed as mean ± SD; a p-value < 0.05 is significant; (*) vs. the control group, (#) vs. the TAA group, and ($) vs. the TAA + SM group all denote a significant difference

The renal assault evoked by TAA resulted in a significant increase in the gene expression levels of TβRI and TβRII (p-value < 0.0001) compared to the control group. Those effects were significantly reversed in both treatment groups (p-value < 0.0001) if compared to the TAA group. Moreover, TβRI showed more reduction with exosome treatment as its expression returned to near control values (p-value > 0.05) if compared to the control group (Fig. 5a and b).

### ELISA expression levels of collagen type I and CTGF proteins

The renal fibrosis evoked by TAA resulted in a significant increase in the protein expression levels of both collagen I and CTGF proteins (p-value < 0.0001) if compared to the control group. All were significantly reversed in both treatment groups (p-values < 0.0001) compared to the TAA group. Moreover, both proteins exhibited more reduction with exosome treatment, as their expression returned to near-control values (p-value > 0.05) if compared to the control group (Fig. 5c and d).

### Western blotting of different SMAD proteins

**Fig. 6:**
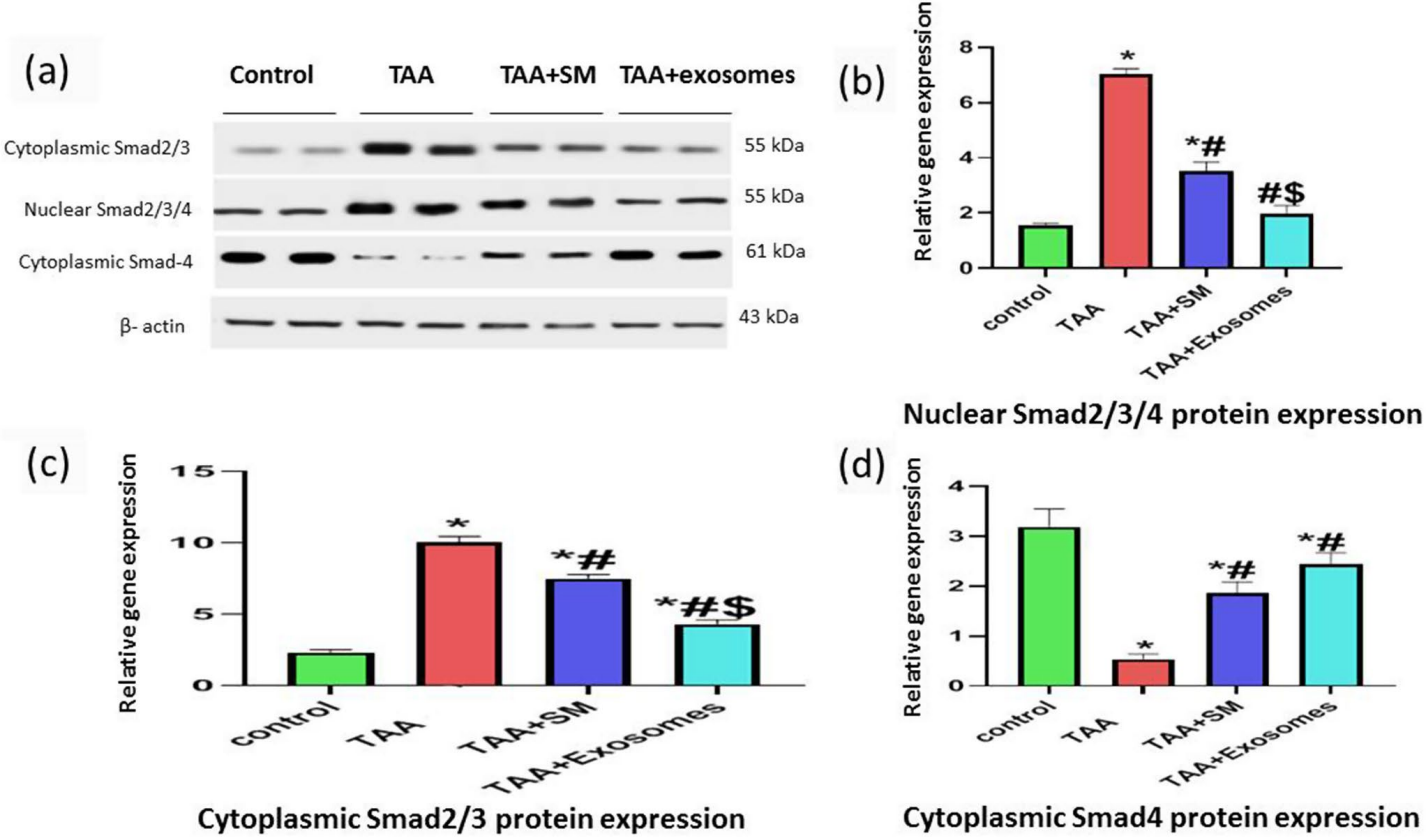
Western analysis of different SMAD proteins. (**a**) Western blotting of nuclear SMAD2/3/4, cytoplasmic SMAD2/3, and cytoplasmic SMAD4 against β-actin, with the molecular weight of the proteins marked; (**b**) expression levels of nuclear SMAD2/3/4 protein; (**c**) expression levels of cytoplasmic SMAD2/3 protein; (**d**) expression levels of cytoplasmic SMAD4 protein. Data are expressed as mean ± SD; p-value < 0.05 is significant; (*) vs. the control group, (#) vs. the TAA group, and ($) vs. the TAA + SM group all denote a significant difference

Both the cytoplasmic SMAD2/3 complex and their translocated proteins to the nucleus after binding SMAD4 (SMAD2/3/4) illustrated significantly increased levels in the TAA group when compared to the control group (p-value < 0.0001). That was reversed considerably in both treatment groups (p-value < 0.0001 if compared to the TAA group) with proximity to control values with the exosomes treatment (p-value of 0.0003‏ for cytoplasmic SMADs and > 0.05 for nuclear ones (Fig. 6a and b, and 6c).

In contrast, the cytoplasmic SMAD4 exhibited significantly decreased levels in the TAA group (p-value < 0.0001). Its expression levels were significantly higher in both treatment groups (p-value < 0.0001) (Fig. 6a and d).

## Discussion

Silymarin, a typical example of plant-based therapy, is known for its potential nephroprotective effects [27, 28]. MSCs have been described as the most widely used type of cell for cell therapy of damaged kidneys [29]. MSC exosomes’ multipotency and self-renewing capabilities make them a promising vehicle for fibrotic kidney diseases. Using exosomes as alternatives for their derived cells is a prodigious step towards cell-free therapeutics [10]. This study aimed to compare silymarin, an example of the phytomedicine category, with MSC exosomes, an example of the regenerative medicine category, to explore whether they have equally promising renoprotective effects.

It is known that no previous research has investigated the roles of either SM or BM-MSC-derived exosomes on TGF-β signaling in TAA-induced kidney damage.

Thioacetamide (TAA) is an ideal model for evaluating anti-fibrotic compounds in experimental animals [4]. It has been extensively linked to chronic and fibrotic kidney diseases, and much literature has described different protective compounds against the nephrotoxicity induced by TAA [30–35]. In the current study, the TAA-induced model illustrated a significant deterioration of kidney functions and significant upregulation of critical markers of the TGF-β/SMAD pathway, including the receptors TβRI and TβRII, the cytoplasmic SMAD2/3 complex, the nuclear SMAD2/3/4, collagen I, and CTGF proteins. This is consistent with an earlier study [36] that demonstrated the upregulation of SMAD2 and SMAD3 in TAA-induced renal assault; such effects were reversed by vanillin. Similar toxic impacts of TAA on the TGF-β1/SMAD pathway were additionally applied to liver tissues [37–41].

This study illustrated significantly decreased levels of the co-SMAD partner SMAD4 in the fibrotic TAA group; it may be utilized during the formation of the hetero-oligomeric complex SMAD2/3/4 with no appropriate induction for its synthesis. SMAD4 has a diverse role; it acts as a fine tuner to promote SMAD3-facilitated fibrosis while inhibiting NF-κB-driven renal inflammation, and its disruption does not alter phosphorylation or translocation of SMAD2/3 [42]. In contrast to the results of this study, the expression of Smad4 was increased in TAA-induced hepatic fibrosis in mice and then alleviated by silymarin [43]. Still, the protein expression levels of other markers (TGF-β1, SMAD2/3, p-SMAD2/3, and collagen-1) align with the research results. The role of SMAD4 seems relatively blurred and remains to be explored.

As for the effects of silymarin and MSCs-exosomes, both succeeded in attenuating kidney fibrosis through the operation of the TGF-β/SMAD signaling pathway through the restoration of the relative levels of expression of TβRI and TβRII genes, cytoplasmic SMAD2/3, nuclear SMAD2/3/4, and cytoplasmic SMAD4 proteins. Worthy noted that the exosomes group proved more significant improvements in all parameters than the SM group, except for the SMAD4 protein.

Concerning SM, findings of a previous study in diabetic kidney injury in rats are following this study [44]; they suggest SM causes the protein expression levels of TGF-β1 and SMAD2/3 to be notably decreased, and SM nanoliposomes co-inhibit JAK2/STAT3 and TGF-β/SMAD signaling pathways. Consistent results were verified in peritoneal fibrosis [45] and liver fibrosis [46–48].

The results regarding BM-MSCs exosomes were supported by Liu et al. [49], as they showed TGF-β1 stimulation increased the expression of collagen-I, which the administration of BM-MSCs exosomes reversed. In addition, Nagaishi et al. [50] detected that these exosomes ameliorated kidney inflammation and TGF-β production.

One of the limitations of this study was the lack of a fifth group (TAA + SM + Exosomes group), which would undoubtedly strengthen the current research.

## Conclusions

The present study delivers new insights into the positive functional roles of silymarin and BM-MSC exosomes in thioacetamide-induced renal fibrosis by inhibiting the TGF-β/SMAD pathway in rats. It presents novel preclinical findings that may apply to humans with renal fibrosis in the future.

## Electronic supplementary material

Below is the link to the electronic supplementary material.

Supplementary Material 1 S1: Supplementary Information 1: TGF-β/SMAD crosstalk pathway, showing the studied biomarkers. (a) TGF-β binding to homodimeric TβRII; (b) Recruitment and phosphorylation of two units of TβRI: (c) TβRI phosphorylates and activates SMAD2/3 and non-SMAD pathways: (d) Formation of a cytosolic hetero-oligomer SMAD2/3/4: (e) the nuclear SMAD2/3/4 control the expression of collagen I and CTGF.

Supplementary Material 2 S2: Summary of the steps of methodology in this study.

Supplementary Material 3 S3: The original western blot results. (a) Western blotting of β-actin (the loading control); (b) Western blotting of cytoplasmic SMAD4 protein; (c) Western blotting of cytoplasmic SMAD2/3 protein; (d) Western blotting of nuclear SMAD2/3/4 protein.

Supplementary Material 4 S4: Masson’s trichrome highlighting peritubual fibrosis in TAA group (x1000).

## Data Availability

All data generated during this work are included in this manuscript.

## Abbreviations

BM-MSCs: Bone Marrow-Mesenchymal Stem Cells
CTGF: Connective Tissue Growth Factor
ECM: Extracellular Matrix
EMT: Endothelial-Mesenchymal Transition
ERK: Extracellular Regulated Protein Kinase
H&E: Hematoxylin–Eosin
I.P: Intraperitoneally
I-Smads: Inhibitory Smads
JNK: C-Jun N-Terminal Kinase
MAPK: Mitogen-Activated Protein Kinase
MMPs: Matrix Metalloproteinases
MSCs: Mesenchymal Stem Cells
R-Smads: Receptor-Regulated Smads
SM: Silymarin
SMADs: Suppressor of Mothers against Decapentaplegic
TAA: Thioacetamide
TGF-β: Transforming Growth Factor-β
TβRI: Type I TGF-β Receptor
TβRII: Type II TGF-β Receptor
