# Supplementary figures and images for: Silymarin and MSC-exosomes ameliorate thioacetamide-evoked renal fibrosis by inhibiting TGF-β/SMAD pathway in rats
Source: Mol Biol Rep. 2024 Apr 18;51(1):529. doi: 10.1007/s11033-024-09343-6 (PMC11026270; doi:10.1007/s11033-024-09343-6)

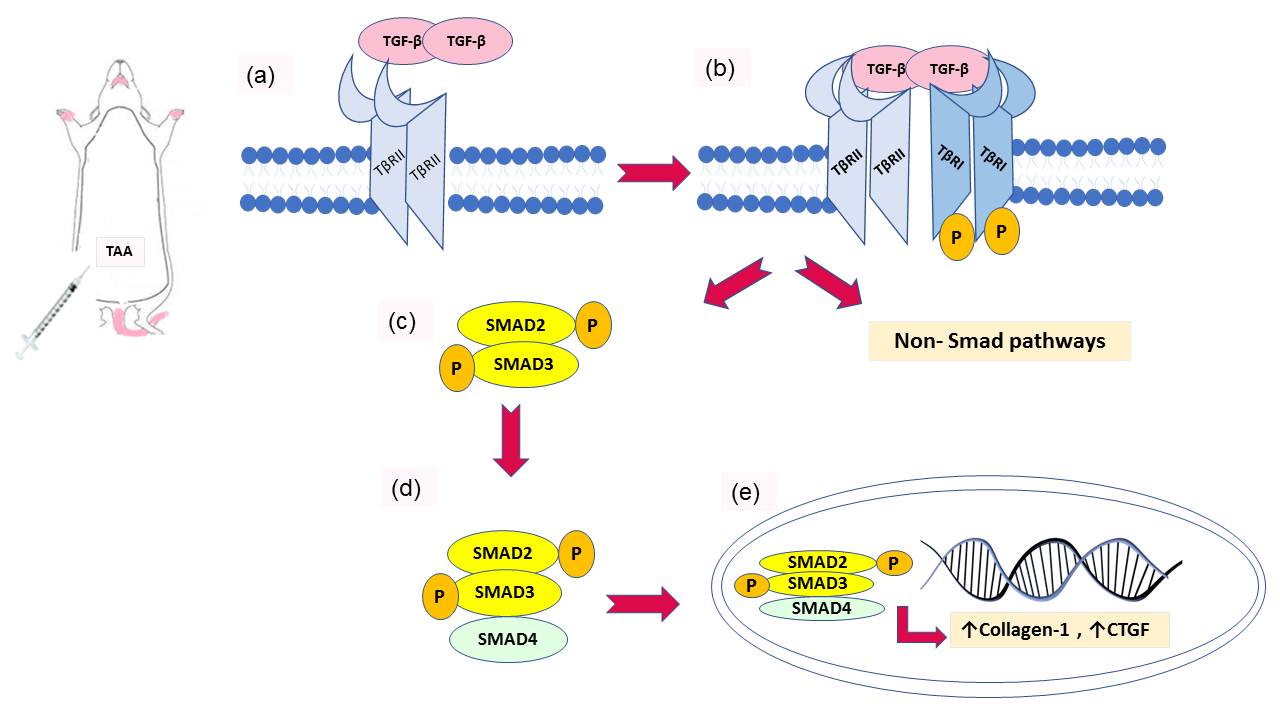

Supplement: Supplementary file 1 — Supplementary Material 1 S1: Supplementary Information 1: TGF-β/SMAD crosstalk pathway, showing the studied biomarkers. (a) TGF-β binding to homodimeric TβRII; (b) Recruitment and phosphorylation of two units of TβRI: (c) TβRI phosphorylates and activates SMAD2/3 and non-SMAD pathways: (d) Formation of a cytosolic hetero-oligomer SMAD2/3/4: (e) the nuclear SMAD2/3/4 control the expression of collagen I and CTGF. [file 11033_2024_9343_MOESM1_ESM.jpg]

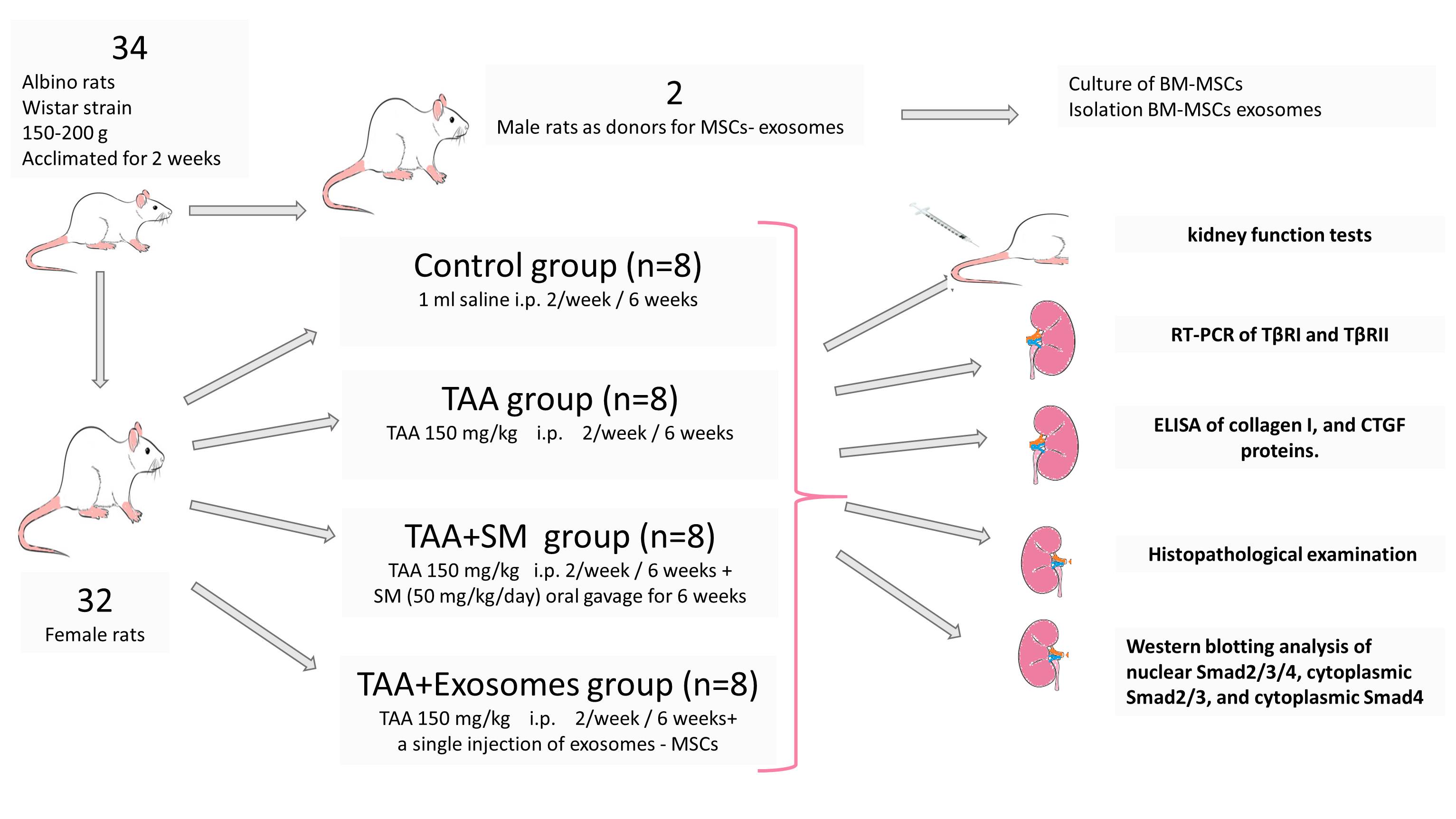

Supplement: Supplementary file 2 — Supplementary Material 2 S2: Summary of the steps of methodology in this study. [file 11033_2024_9343_MOESM2_ESM.jpg]

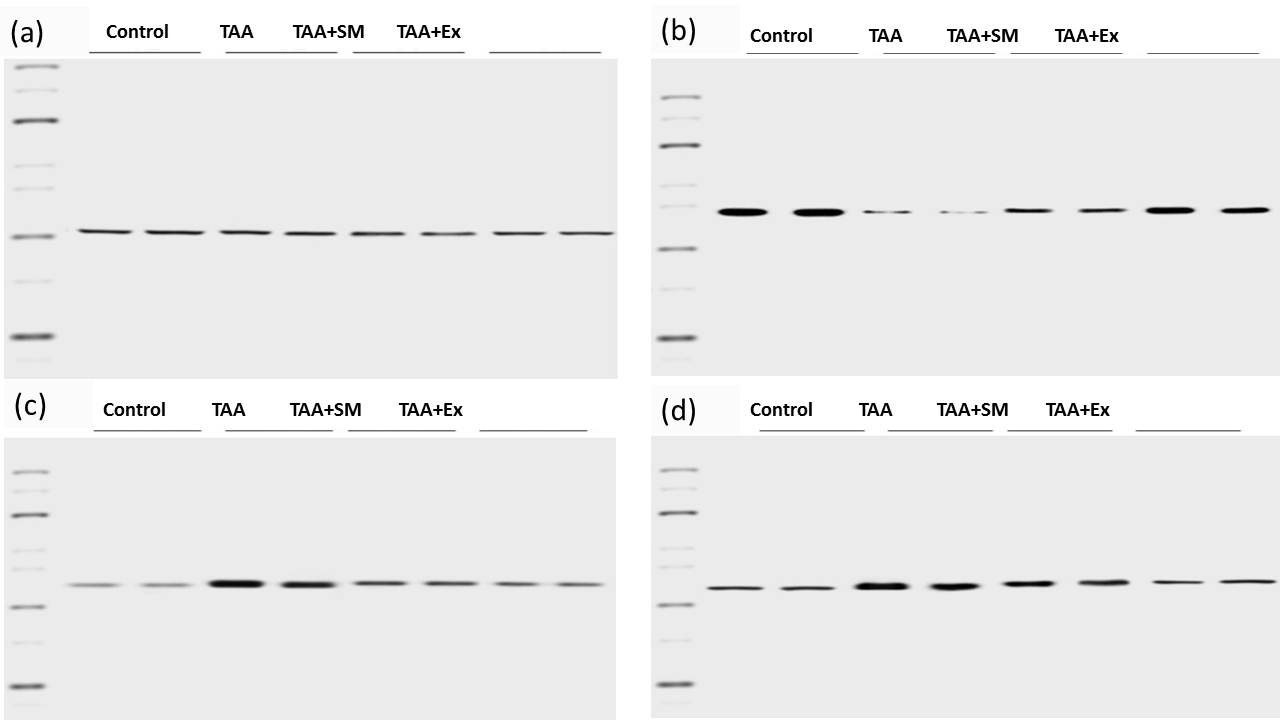

Supplement: Supplementary file 3 — Supplementary Material 3 S3: The original western blot results. (a) Western blotting of β-actin (the loading control); (b) Western blotting of cytoplasmic SMAD4 protein; (c) Western blotting of cytoplasmic SMAD2/3 protein; (d) Western blotting of nuclear SMAD2/3/4 protein. [file 11033_2024_9343_MOESM3_ESM.jpg]

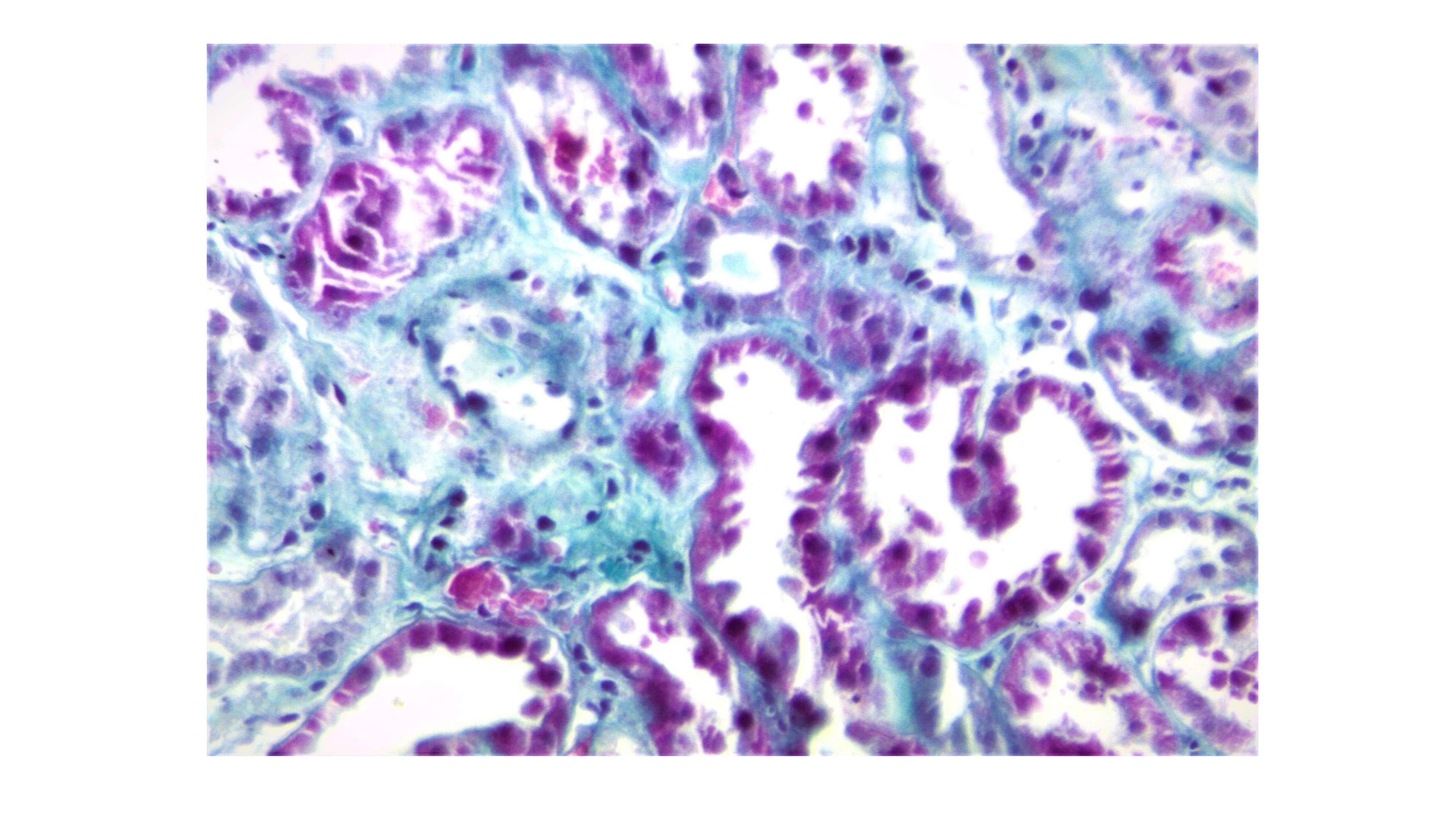

Supplement: Supplementary file 4 — Supplementary Material 4 S4: Masson’s trichrome highlighting peritubual fibrosis in TAA group (x1000). [file 11033_2024_9343_MOESM4_ESM.jpg]
